# Predicted lean body mass in relation to cognitive function in the older adults

**DOI:** 10.3389/fendo.2023.1172233

**Published:** 2023-07-06

**Authors:** Hong-Jian Gong, Xingyao Tang, Yin-He Chai, Yu-Shun Qiao, Hui Xu, Ikramulhaq Patel, Jin-Yan Zhang, Jian-Bo Zhou

**Affiliations:** ^1^ Department of Endocrinology, Beijing Tongren Hospital, Capital Medical University, Beijing, China; ^2^ Beijing Tongren Hospital, Capital Medical University, Beijing, China

**Keywords:** predicted lean mass, cognitive function, older adults, cross-sectional study, information processing speed

## Abstract

**Background:**

Previous findings about lean body mass (LBM) and cognitive function remain unclear. We aimed to examine this association by using data from the National Health and Nutrition Examination Survey (NHANES).

**Methods:**

Using data from the NHANES 2011-2014, we conducted logistic regression models to investigate the relation between the predicted LBM and domain-specific cognitive function assessed by Digit Symbol Substitution Test (DSST), Consortium to Establish a Registry for Alzheimer’s Disease Word Learning test (CERAD-WL) and Delayed Recall test (CERAD-DR), and Animal Fluency (AF) for information processing speed, memory, and executive function, respectively. Cognitive impairment was defined as the lowest quartile of each cognitive test in the total population. Sex-stratified analysis was further made.

**Results:**

A total of 2955 participants aged 60 and above (mean [SD] age, 69.17[0.20] years; 1511 female [51.13%]) were included in the study. After being adjusted for social economic factors, anthropometric parameters, and diseases, we found a positive association between predicted LBM and information processing speed (Odds ratio of DSST impairment= 0.95, 95%CI= 0.91 to 0.99) regardless of body mass index and sex. Compared with patients in the first quartile of predicted LBM, those in the fourth quartile had an odds ratio of 0.355 (95% confidence interval 0.153-0.822) for DSST impairment. No significant relation in other cognitive tests and predicted LBM was found whether stratified by sex or not.

**Conclusion:**

Our findings point to the association between predicted lean body mass and cognitive dysfunction in information processing speed, which could be used for early detection and prevention of deterioration of cognitive function among older adults.

## Introduction

Mild cognitive impairment (MCI), characterized by subtle changes in memory and thinking, is thought to be a reversible stage between being cognitively unimpaired and dementia (1). Approximately 12% to 18% of people aged 60 or older have MCI (1), of which 10% to 15% will progress to dementia each year (2–4), causing an enormous economic burden. In 2022, estimated total payments for all individuals with Alzheimer’s or other dementias reach $321 billion (1). Because there are no disease-modifying methods (5), reducing the more modifiable risk of developing cognitive impairment or dementia is of high priority, especially in our rapidly aging population (6).

Some studies have linked body mass index (BMI) with a change in cognitive function (7–9). Higher BMI is thought to be a risk factor in middle age (10, 11). And due to lifestyle changes associated with incipient cognitive impairment, a more steep decline may be seen in BMI with a high (vs low) burden of AD or cerebral vascular disease (12). However, as a combination of fat mass and lean body mass (LBM), BMI may not adequately capture the differences in body composition. Older adults tend to have more fat mass and less muscle mass (lean body mass) with BMI unchanged (13, 14). Therefore, BMI may not be able to discriminate individuals at risk of cognitive dysfunction correctly. Fat mass was found higher in cognitively intact people compared with those not with covered mechanisms (15). However, the reported results about the association between body composition with overall cognitive function are controversial (16–18), and there is a paucity of data examining the relationship between LBM with specific cognitive domains (19), which is important to understand the relation between body composition and cognition.

Therefore, exploring the independent role of predicted LBM related to specific cognitive function in adults aged 60 or above may improve our knowledge of body composition and cognitive function and help to find the people with a high possibility of worse cognitive function.

## Methods

### Study population

The National Health and Nutrition Examination Survey (nhanes) is a series of continuous, ongoing cross-sectional surveys (20). Representative samples of the civilian noninstitutionalized household population of the United States were selected by a complex, multistage probability sampling design (20). Data were collected by personal interview, mobile physical examination, and laboratory tests and were released after every 2-year cycle. In this study, we incorporated data from two cycles of the NHANES (2011–2014) phases during which cognitive function tests were conducted.

### Exposure measurements

Instead of direct methods of detecting lean body mass, we use validated anthropometric prediction equations to calculate predicted lean mass developed based on the populations of the NHANES 1999–2006 because of the cost of money and time (21). A total of 7531 men and 6534 women who underwent dual-energy X-ray absorptiometry (DXA) examination were included in this database, which is complex multistage probability sampled (21). Briefly, sex-separated analyses were conducted. DXA-measured lean body mass was predicted as a dependent variable about different combinations of anthropometric measures including age, ethnicity, height (cm), weight (kg), BMI (kg/m^2^), waist circumference (cm), other circumference measures (i.e. arm, calf, and thigh (cm)) and skinfold measures (i.e. triceps and subscapular (mm)). The most accurate model used for prediction was determined in the prediction group and further validated predicted values in an independent group. By comparing predicted scores with the DXA-measured values and their correlation with obesity-related biomarkers, the predicted equation proved a high predictive ability for LBM (men: R^2 ^= 0.91; women: R^2 ^= 0.85). We calculated the predicted value of lean body mass according to the equation in the former study (21, 22).

### Outcomes

Cognitive function was examined by a series of cognitive function tests conducted on all respondents aged 60 years and older in a mobile examination center (23), including the Digit Symbol Substitution Test (DSST), Consortium to Establish a Registry for Alzheimer’s Disease Word Learning test (CERAD-WL) and Delayed Recall test (CERAD-DR), and Animal Fluency (AF). These tests evaluated domains of information processing speed (DSST), memory (CERAD), and executive function (AF).

In the DSST, participants are asked to fill 133 boxes according to symbols that were paired to nine numbers within 2 minutes (24). The score is the total number of correct matches. In the NHANES, participants were shown how to perform the task and then filled several practice boxes before the test.

The CERAD Word Learning subtest includes immediate and delayed learning parts, using a word list of 10 unrelated words (25). In the NHANES, participants were requested to read each word in the list aloud and recall as many as possible immediately. This process was repeated three times with the order of words changed (CERAD-WL -score1, CERAD-WL -score2, CERAD-WL-score3) and the total score for the learning task was 30. The delayed recall trial was conducted after approximately 8-10 minutes, where participants were requested to recall the words used in the CERAD-WL trial without review of the word list. CERAD-WL refers to the sum of the four scores, and CERAD-DR refers to the last score.

In the AF test, participants are asked to name as many animals as possible in one minute. The score is the sum of the number of correct answers. In NHANES, participants first were asked to name three items of clothing, another verbal fluency category, as a practice test.

Because there is no gold standard regarding the threshold score for which the cognitive tests indicate cognitive impairment, we selected the lowest quartile in the study group (DSST ≤ 34 points, CERAD-DR ≤ 4 points, CAEDR-WL ≤ 20 points, AF ≤ 13 points) to indicate poor cognitive performance, or impairment, consistent with methods previously published in the literature (23, 26).

### Statistical analysis

Demographic variables were presented as means as the mean (standard deviation (SD)) for continuous variables or as the number of participants (percentage) for categorical variables according to the LBM. One-way analysis of variance (ANOVA) and X^2^ test were performed for the comparison of characteristics according to quintiles of predicted lean mass for continuous variables and categorical variables respectively. Logistic regression models were used to calculate ORs and 95% confidence intervals (CIs) for the associations between predicted LBM and cognitive impairment. LBM was first considered as a continuous variable and then categorized into quartiles. We adjusted for age, sex, race/ethnicity (Mexican American, non-Hispanic black, non-Hispanic white, other Hispanic, other race-including multi-Racial), education (college and above, middle and high school, primary school and less), annual-household-income in model 1. We further adjusted for potential mediators, including drinking status (never, former, and current drinker), BMI, hypertension, smoking status (never, former, and current smoker), cardiovascular disease, diabetes, and chronic kidney disease in model 2. We conducted stratified analyses of the association of predicted LBM with cognitive function according to gender. All analyses were performed using R version 4.2.1(http://www.r-project.org). The statistical tests were two-sided, and a P value <0.05 was considered statistically significant.

## Results

**Figure 1:**
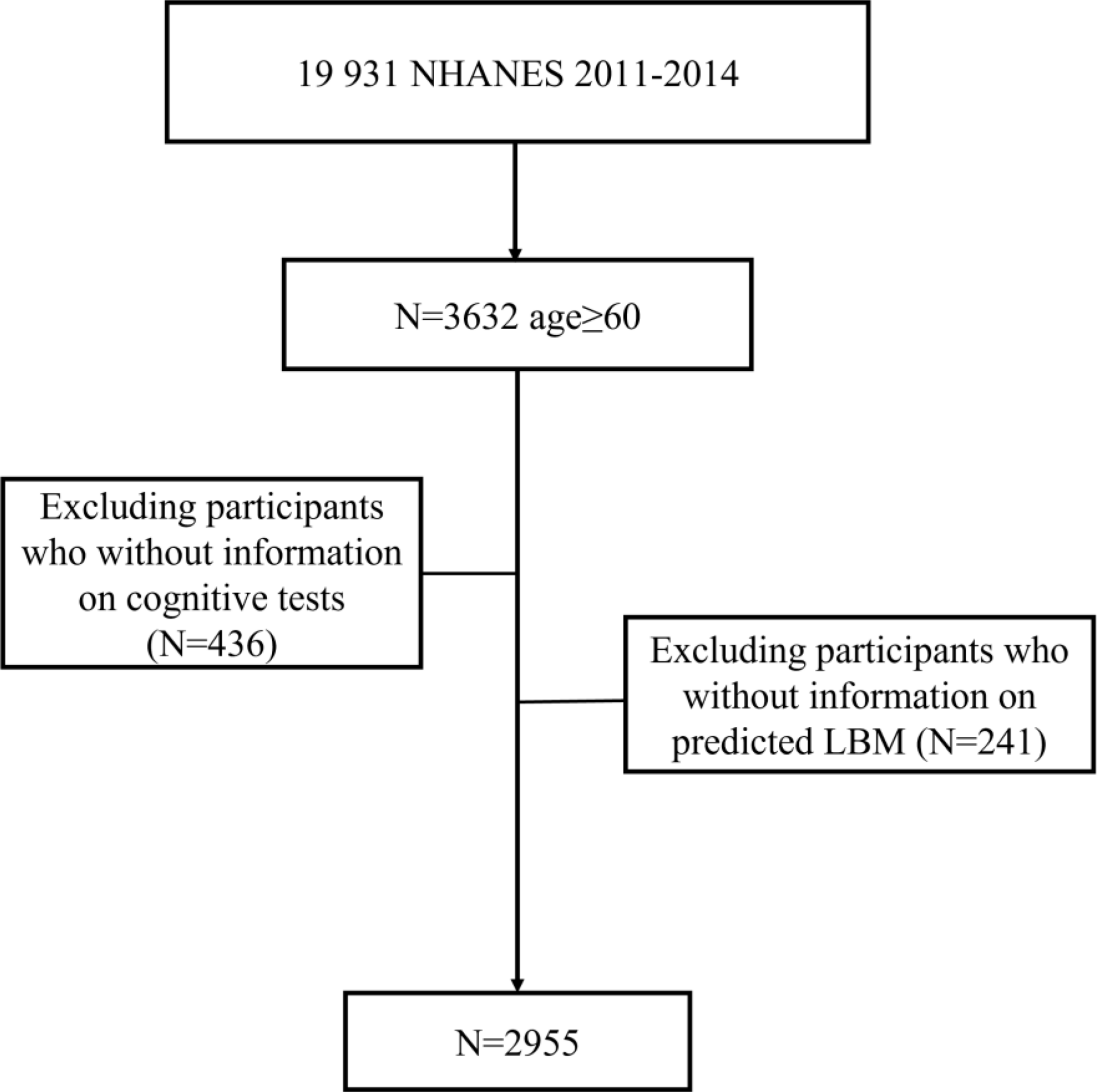
Flow chart of inclusion and exclusion of study participants.

Our analysis was restricted to persons who were ≥ 60 years (n = 3632). We excluded 436 missing information on cognitive tests and additionally 241 missing information on LBM. Therefore, a total of 2955 participants were enrolled in our present analysis (**Figure 1**). **Table 1** depicts the characteristics of participants according to predicted LBM quartiles. The mean age of the study population was 69.17 (SD: 0.20) years. Participants with higher levels of predicted LBM tended to be younger, have higher BMI, higher annual household income, better education, and a higher prevalence of diabetes, and CVD, and were more likely to be male, alcohol consumer, and Non-Hispanic White.

**Table 1:** Characteristics of study participants according to quintiles of predicted lean body mass ^a^.

variable	total	Q1	Q2	Q3	Q4	P trend
Age, mean (SD), years	69.17 (0.20)	71.25 (0.35)	69.13 (0.29)	68.84 (0.32)	67.53 (0.28)	<0.0001
Sex, No. (%)						<0.0001
Female	1511 (51.13)	721 (98.60)	539 (83.44)	205 (31.22)	46 (5.78)	
Male	1444 (48.87)	18 (1.40)	199 (16.56)	534 (68.78)	693 (94.22)	
Ethnicity, No. (%)						<0.0001
Mexican American	274 (9.27)	76 (4.20)	64 (3.52)	77 (4.24)	57 (2.75)	
Non-Hispanic Black	717 (24.26)	68 (4.13)	182 (10.32)	210 (11.28)	257 (9.69)	
Non-Hispanic White	1345 (45.52)	356 (76.64)	320 (75.57)	306 (75.79)	363 (83.86)	
Other Hispanic	315 (10.66)	96 (5.37)	95 (4.88)	81 (4.07)	43 (1.82)	
Other Race-Including Multi-Racial	304 (10.29)	143 (9.67)	77 (5.71)	65 (4.63)	19 (1.88)	
Annual household income, No. (%)						<0.0001
<$65,000	2088 (74.07)	553 (71.85)	536 (67.23)	515 (61.02)	484 (55.13)	
≥$65,000	731 (25.93)	151 (28.15)	164 (32.77)	186 (38.98)	230 (44.87)	
Education, No. (%)						0.03
College and above	1475 (49.97)	363 (56.18)	361 (59.61)	358 (62.73)	393 (65.74)	
Middle and high school	1105 (37.43)	262 (35.28)	290 (34.62)	276 (30.21)	277 (30.02)	
Primary school and less	372 (12.6)	114 (8.54)	85 (5.76)	105 (7.06)	68 (4.24)	
DM, No. (%)	991 (33.54)	176 (18.80)	255 (28.05)	260 (28.61)	300 (33.05)	<0.001
CKD, No. (%)	985 (34.93)	242 (33.81)	253 (30.78)	244 (32.26)	246 (30.17)	0.62
CVD, No. (%)	641 (21.7)	128 (17.09)	156 (18.81)	178 (23.54)	179 (26.59)	0.01
Hypertension, No. (%)	2103 (71.17)	511 (66.52)	535 (67.10)	511 (64.16)	546 (69.92)	0.38
Smoke, No. (%)						<0.0001
former	1106 (37.45)	165 (28.99)	236 (31.88)	324 (44.78)	381 (51.92)	
never	1468 (49.71)	495 (60.53)	385 (54.00)	313 (44.99)	275 (38.76)	
now	379 (12.83)	78 (10.48)	117 (14.12)	101 (10.23)	83 (9.31)	
Alcohol, No. (%)						<0.0001
former	803 (27.61)	161 (21.66)	202 (23.30)	201 (23.43)	239 (24.58)	
never	492 (16.92)	214 (21.48)	130 (15.09)	89 (9.08)	59 (7.58)	
now	1613 (55.47)	349 (56.86)	392 (61.60)	441 (67.49)	431 (67.84)	
BMI, mean (SD), kg/m^2^	28.97 (0.20)	24.61 (0.17)	29.24 (0.19)	29.34 (0.40)	32.51 (0.47)	<0.0001
WC, mean (SD), cm	102.38 (0.49)	89.20 (0.53)	101.13 (0.42)	104.10 (0.75)	114.50 (0.90)	<0.0001
Lean body mass, mean (SD), kg/m^2^	48.65 (0.35)	34.80 (0.15)	42.83 (0.11)	51.40 (0.12)	64.69 (0.54)	<0.0001
CERAD-WL score, mean (SD), score	25.90 (0.31)	26.04 (0.37)	26.19 (0.43)	25.72 (0.32)	25.64 (0.46)	0.63
CERAD-DR score, mean (SD), score	6.23 (0.09)	6.30 (0.11)	6.27 (0.14)	6.21 (0.11)	6.16 (0.16)	0.77
AF score, mean (SD), score	18.14 (0.18)	17.17 (0.27)	17.96 (0.33)	18.41 (0.37)	18.97 (0.35)	<0.0001
DSST score, mean (SD), score	52.60 (0.57)	51.63 (0.92)	54.24 (0.91)	51.30 (0.88)	53.10 (0.72)	0.05

AF, animal fluency test; CERAD-DR, Consortium to Establish a Registry for Alzheimer’s Disease Delayed Recall test; CERAD-WL, Consortium to Establish a Registry for Alzheimer’s Disease Word Learning test; DSST, Digit Symbol Substitution Test; BMI, body mass index; CI, confidence interval; Ref, reference; Edu, education; Eth, Ethnicity; DM, diabetes mellitus; CVD, cardiovascular disease; CKD, chronic kidney disease.

a All estimates accounted for sample weights and complex survey designs, and means and percentages were adjusted for survey weights of NHANES.

When analyzed as a continuous variable, higher LBM was associated with a lower risk of DSST impairment (OR= 0.97, 95%CI= 0.94 to 1.00) (**Table 2**), while aging was associated with higher risk (OR= 1.11, 95%CI= 1.08 to 1.14). Compared to Mexican American, Non-Hispanic White has a lower risk of DSST impairment, while Non-Hispanic Black has a higher risk. Participants with higher education, and higher annual household income is less likely to develop DSST impairment. When adjusting for BMI, hypertension, smoke, alcohol, CVD, DM, and CKD, the association of LBM with DSST strengthened (OR= 0.95, 95%CI= 0.91 to 0.99). However, lean mass was not associated with CERAD-WL (OR=0.97, 95%CI= 0.94 to 1.01), CERAD -DR (OR= 0.98, 95%CI= 0.95 to 1.01), and AF (OR= 0.99, 95%CI= 0.96 to 1.02) impairment after adjusting for confounders (**Supplementary Table 1**).

**Table 2:** Odds ratio (95%CI) for the associations between lean body mass and DSST impairment (results of model 2) ^ac^ .

Characteristic	Forest pot	OR (95% CI)
Lean body mass, kg/m^2^	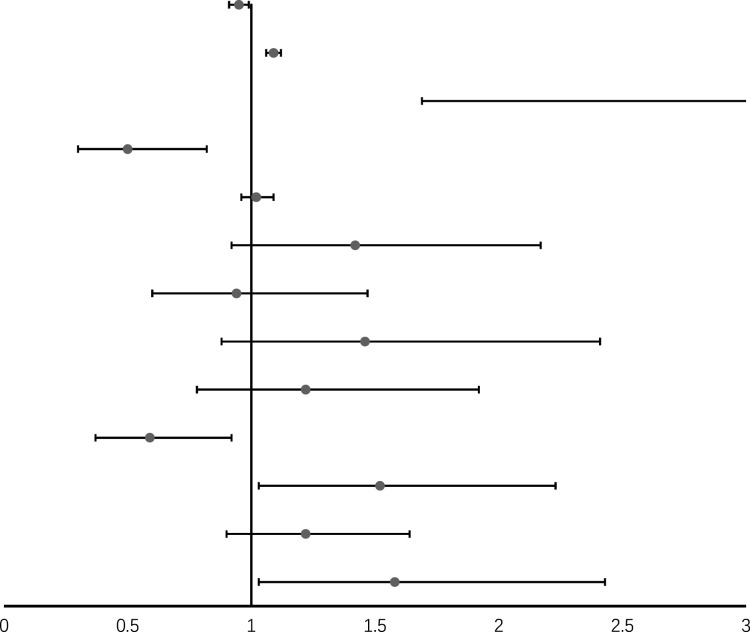	0.95 (0.91, 0.99) *
Age, years		1.09 (1.06, 1.12) *
Sex, male vs female		3.71 (1.69, 8.12) *
Annual household income ≥$65,000 vs <$65,000		0.50 (0.30, 0.82) *
BMI, kg/m^2^		1.02 (0.96, 1.09)
Hypertension		1.42 (0.92, 2.17)
Former smoker vs never		0.94 (0.60, 1.47)
Now smoker vs never		1.46 (0.88, 2.41)
Former drinker vs never		1.22 (0.78, 1.92)
Now drinker vs never		0.59 (0.37, 0.92) *
CVD		1.52 (1.03, 2.23) *
DM		1.22 (0.90, 1.64)
CKD		1.58 (1.03, 2.43)

AF, animal fluency test; CERAD-DR, Consortium to Establish a Registry for Alzheimer’s Disease Delayed Recall test; CERAD-WL, Consortium to Establish a Registry for Alzheimer’s Disease Word Learning test; DSST, Digit Symbol Substitution Test; BMI, body mass index; CI, confidence interval; Ref, reference; Edu, education; Eth, Ethnicity; DM, diabetes mellitus; CVD, cardiovascular disease; CKD, chronic kidney disease.

a All estimates accounted for sample weights and complex survey designs, and means and percentages were adjusted for survey weights of NHANES.

^C^ Model 2 was adjusted for age, sex, race/ethnicity (Mexican American, non-Hispanic black, non-Hispanic white, other Hispanic, other race-including multi-Racial), education (college and above, middle and high school, primary school and less), annual-household-income, drinking status (never, former, and current drinker), BMI, hypertension, smoking status (never, former, and current smoker), cardiovascular disease, diabetes, and chronic kidney disease.

* p<0.05.

When the predicted LBM was considered as a categorical variable, people with higher predicted LBM were more likely to have a lower risk of DSST impairment. (p for trend =0.018) and the highest predicted LBM quartile was associated with a 65.5% decrease in risk of DSST impairment (OR=0.355, 95%CI= 0.153 to 0.822) (**Table 3**). While the risk of CERAD-WL, CERAD-DR, and AF impairment was not associated with the category of predicted LBM (**Supplementary Table 2**).

**Table 3:** Odds ratio (95%CI) for the associations between lean body mass and DSST impairment ^b^, by predicted lean body mass index quartiles ^a^.

DSST impairment	Lean body mass							
	Q1	Q2	P	Q3	P	Q4	P	p for trend
Crude model	ref	0.74 (0.56,0.97)	0.04	0.82 (0.61,1.11)	0.19	0.57 (0.40,0.80)	0.00	0.01
Model 1	ref	0.63 (0.37, 1.08)	0.09	0.47 (0.22, 1.03)	0.06	0.37 (0.15, 0.90)	0.03	0.03
Model 2	ref	0.64 (0.38, 1.07)	0.08	0.47 (0.21, 1.02)	0.06	0.3 6(0.15, 0.82)	0.02	0.02

AF, animal fluency test; CERAD-DR, Consortium to Establish a Registry for Alzheimer’s Disease Delayed Recall test; CERAD-WL, Consortium to Establish a Registry for Alzheimer’s Disease Word Learning test; DSST, Digit Symbol Substitution Test; BMI, body mass index; CI, confidence interval; Ref, reference; Edu, education; Eth, Ethnicity; DM, diabetes mellitus; CVD, cardiovascular disease; CKD, chronic kidney disease.

a All estimates accounted for sample weights and complex survey designs, and means and percentages were adjusted for survey weights of NHANES.

b DSST impairment was defined as a socre ≤ 34 points.

When we examined the association between LBM and cognitive function stratified by sex, predicted LBM was associated with a lower risk of DSST impairment only in females (OR=0.9, 95%CI= 0.84 to 0.96) (**Table 4**). While taken as a categorical variable, higher predicted LBM was negatively associated with DSST impairment in both genders (female: OR=0.193, 95%CI= 0.043 to 0.876; male: OR=0.239, 95%CI= 0.043 to 0.863) (**Supplementary Table 4**).

**Table 4:** Odds ratio (95%CI) for the associations between lean body mass and DSST impairment, stratified by sex (results of model 2) ^ac^.

Character	Forest plot	OR (95% CI)	
		Female	Male
Lean body mass, kg/m^2^	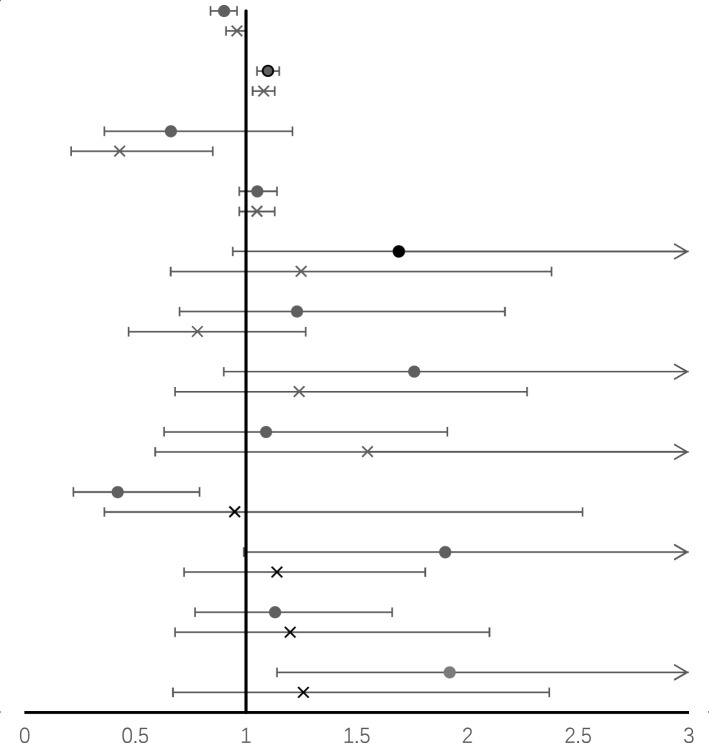	0.90 (0.84, 0.96) *	0.96 (0.91, 1.00)
Age, years		1.10 (1.05, 1.15) *	1.08 (1.03, 1.13) *
Annual household income ≥$65,000 vs <$65,000		0.66 (0.36, 1.21)	0.43 (0.21, 0.85) *
BMI, kg/m^2^		1.05 (0.97, 1.14)	1.05 (0.97, 1.13)
Hypertension		1.69 (0.94, 3.04)	1.25 (0.66, 2.38)
Former smoker vs never		1.23 (0.70, 2.17)	0.78 (0.47, 1.27)
Now smoker vs never		1.76 (0.90, 3.47)	1.24 (0.68, 2.27)
Former drinker vs never		1.09 (0.63, 1.91)	1.55 (0.59, 4.11)
Now drinker vs never		0.42 (0.22, 0.79) *	0.95 (0.36,2.52)
CVD		1.90 (0.99, 3.65)	1.14 (0.72, 1.81)
DM		1.13 (0.77, 1.66)	1.20 (0.68, 2.10)
CKD		1.92 (1.14, 3.23) *	1.26 (0.67, 2.37)

AF, animal fluency test; CERAD-DR, Consortium to Establish a Registry for Alzheimer’s Disease Delayed Recall test; CERAD-WL, Consortium to Establish a Registry for Alzheimer’s Disease Word Learning test; DSST, Digit Symbol Substitution Test; BMI, body mass index; CI, confidence interval; Ref, reference; Edu, education; Eth, Ethnicity; DM, diabetes mellitus; CVD, cardiovascular disease; CKD, chronic kidney disease.

a All estimates accounted for sample weights and complex survey designs, and means and percentages were adjusted for survey weights of NHANES.

^C^ Model 2 was adjusted for age, sex, race/ethnicity (Mexican American, non-Hispanic black, non-Hispanic white, other Hispanic, other race-including multi-Racial), education (college and above, middle and high school, primary school and less), annual household income, drinking status (never, former, and current drinker), BMI, hypertension, smoking status (never, former, and current smoker), cardiovascular disease, diabetes, and chronic kidney disease.

* p<0.05.

## Discussion

Our analysis of 2 cycles of the NHANES study showed that those with higher predicted LBM were associated with a lower risk of DSST impairment, where no association was found between BMI and DSST impairment. Although there was a trend showing that higher lean body mass was associated with a lower risk of cognitive impairment in other tests, we did not find statistical significance between groups.

Few investigators have studied the relationship between body composition and specific cognitive functions in normal people. A recent study reported uncorrelated associations between LBM with psychomotor function, attention, visual learning, and working memory (19). Skeletal muscle mass of the four limbs was associated only with delayed memory in Serena Low’s study, while lower LESM (calculated as added left and right lower limbs divided by square of height) was independently associated with reduced cognitive function globally and specifically in domains of immediate memory, delayed memory and visuospatial/constructional ability (27). To date, some studies have examined the relationship between lean mass loss and overall cognition, with inconsistent conclusions. Our findings are consistent with previous cohort studies that have reported a positive association between LBM and cognitive function (17, 28, 29). In a study of US elders using a standardized psychometric battery, accelerated loss of LBM was associated with worse cognitive performance and structure change of the brain (17). However, in a prospective study to assess various sarcopenia markers in conjunction with cognitive decline, muscle mass was not associated with the progression of cognitive impairment (30). Hye-Mi Noh et al. found that not the group with the highest total LBM but the second in females was associated with a lower risk for cognitive impairment. Discrepant findings may be due to the differences in the tools to screen for cognitive impairment, part of which is the low sensitivity of cognitive impairment; race/ethnicity, or residual confounding. Compared with these studies, NHANES is well designed with higher representativity and less sampling error; and this study included more participants and further stratified with sex and different cognitive tests. In our cohort, the 4th quartile of LBM was associated with higher BMI. However, the association between DSST and LBM remained significant after adjusting for BMI.

The positive association may result from shared mechanisms: lifestyle risk factors and poor nutrition (31). Aging is in conjunction with a sequence of exercise-related changes. Less physical activity can directly attribute to the decline of lean mass: age-related reductions in physical activity is the most important external cause of sarcopenia in normal aging (32). Physical activity can slow down the decline of cognitive function and protect the brain structure (33–36). On the other hand, the decrease in physical activity can also be the result of cognitive impairment, like AD, Parkinson, etc. Studies had found that low DSST was significantly associated with gait speed (37, 38). So, it might be a vicious circle in which physical activity and cognitive dysfunction favor each other. However, considering that dementia is a slowly progressive illness for which clinical symptoms may appear 20 years or more after pathophysiological changes in the brain, the relation between physical activity and cognitive dysfunction needs further explore.

Although this association was only found in the DSST test, the meaning cannot be ignored. DSST is a sensitive test to identify cognitive dysfunction, especially in impairments in processing speed, executive functioning, and working memory (39). The prevalence of low DSST was high (11%) even in the population of well-functioning older adults and related to a higher risk for mortality and disability (26). Participants with low DSST performance had an increased risk of incident all-type dementia (40). And probable pathology basis was declaimed recently that declining processing speeds (tested by DSST) were associated with emerging PET-detected AD pathology in clinically normal older adults (41). In Caterina et al’ work about cognitive-health people, lower DSST was associated with nearly twice the odds of developing 1+ clinical or subclinical disorders of cognition, mobility, and mood (42). Except for cognitive disorders, higher DSST was associated with a 28%-34% lower mortality risk in elders with white matter hyperintensities (43). In this context, this study has its merit in detecting people at high risk of low cognitive function and accepting early multi-domain preventive interventions, thereby interrupting the vicious circles and preventing or delaying dementia onset.

The current study has several limitations to be considered. First, it’s a cross-sectional study, which prevents concluding on the causality between body composition and cognitive function. Second, our study was based on predicted body composition, which is a compromise of accuracy and cost and will inevitably cause measurement errors. However, the predictive ability of anthropometric equations was proved to be high (men: R ^2 ^= 0.91; women: R ^2 ^= 0.85) in an independent large validation study (21). Third, we didn’t discriminate against the lean mass of different regions. Fourth, although we controlled the results for several potential confounders, some variables like APOE level, physical activity, the severity of different diseases, medicine or information on insulin-dependent (or not) were not included, which may have affected the association between LBM and cognitive impairment.

## Conclusion

Our study provides new insights into the body composition and cognition function that predicted LBM was associated with lower DSST regardless of BMI. These findings highlight the importance of monitoring predicted LBM regularly among older adults through simple equations, which may help to identify populations at high risk of cognitive dysfunction for in-time intervention to improve prognosis. Although the mechanisms under this association are not figured out, maintaining relatively higher levels of LBM is importance for older adults. Further research is required to examine the causality and mechanisms between LBM and cognitive function.

## Data availability statement

The original contributions presented in the study are included in the article/**Supplementary Material**. Further inquiries can be directed to the corresponding author.

## Author contributions

H-JG and XT contributed equally as co-first (or last) authors. J-BZ is corresponding author. H-JG, XT, Y-HC, Y-SQ, HX, IP, J-YZ have full access to all the data in this study and take full responsibility as guarantors for the integrity of the data and the accuracy of the data analysis. H-JG and XT contributed to studies selection, data extraction, data analyses, and manuscript drafting. XT, Y-HC, Y-SQ, HX, IP, J-YZ contributed to data analyses, data interpretation, and manuscript drafting. J-BZ, H-JG, XT contributed to study design, data interpretation, and final approval of the manuscript. The corresponding author attests that all listed authors meet authorship criteria and that no others meeting the criteria have been omitted. All authors contributed to the article and approved the submitted version.
