# Supplementary material for: Predicted lean body mass in relation to cognitive function in the older adults
Source: Front Endocrinol (Lausanne). 2023 Jul 6;14:1172233. doi: 10.3389/fendo.2023.1172233 (PMC10358760; doi:10.3389/fendo.2023.1172233)
Supplement: Supplementary file 1 [file Table_1.docx]

**Supplement Materials**

**Supplementary tables**

Table 1 Odds ratio (95%CI) for the associations between lean body mass and four cognitive tests ^a^

Table 2 Odds ratio (95%CI) for the associations between lean body mass and cognitive tests, by predicted lean body mass index quartiles ^a^

Table 3 Odds ratio (95%CI) for the associations between lean body mass and four cognitive tests, stratified by sex ^a^

Table 4 Odds ratio (95%CI) for the associations between lean body mass and cognitive tests separated by sex, by predicted lean body mass index quartiles ^a^

Abbreviations: AF, animal fluency test; CERAD-DR, Consortium to Establish a Registry for Alzheimer’s Disease Delayed Recall test; CERAD-WL, Consortium to Establish a Registry for Alzheimer’s Disease Word Learning test; DSST, Digit Symbol Substitution Test; BMI, body mass index; CI, confidence interval; Ref, reference; Edu, education; Eth, Ethnicity; DM, diabetes mellitus; CVD, cardiovascular disease; CKD, chronic kidney disease.

^a^ All estimates accounted for sample weights and complex survey designs, and means and percentages were adjusted for survey weights of NHANES.

^C^ Model 1 was adjusted for age, sex, race/ethnicity (Mexican American, non-Hispanic black, non-Hispanic white, other Hispanic, other race-including multi-Racial), education (college and above, middle and high school, primary school and less), annual-household-income.

^d^ Model 2 was further adjusted for drinking status (never, former, and current drinker), BMI, hypertension, smoking status (never, former, and current smoker), cardiovascular disease, diabetes, and chronic kidney disease.

* p＜0.05

Table 1 Odds ratio (95%CI) for the associations between lean body mass and four cognitive tests ^a^

|  | DSST impairment | CARED-WL impairment | CARED-DR impairment | AF impairment |
| --- | --- | --- | --- | --- |
| Model 1^c^ | 0.97 (0.94, 1.00) * | 0.98 (0.96,1.00) * | 0.99 (0.98,1.01) | 0.99 (0.98,1.01) |
| Model 2^d^ | 0.95 (0.91, 0.99) * | 0.97 (0.94,1.01) | 0.98 (0.95,1.01) | 0.99 (0.96,1.02) |

Table 2 Odds ratio (95%CI) for the associations between lean body mass and cognitive tests, by predicted lean body mass index quartiles ^a^

| Lean body mass | DSST impairment | | CARED-WL impairment | | CARED-DR impairment | | AF impairment | |
| --- | --- | --- | --- | --- | --- | --- | --- | --- |
|  | OR (95%CI) | p | OR (95%CI) | p | OR (95%CI) | p | OR (95%CI) | p |
| Q1 | ref |  | ref |  | ref |  | ref |  |
| Q2 | 0.64(0.38, 1.07) | 0.08 | 1.24(0.76, 2.03) | 0.36 | 1.25(0.79,1.98) | 0.31 | 0.97(0.61,1.55) | 0.90 |
| Q3 | 0.47(0.21, 1.02) | 0.06 | 0.87(0.44, 1.72) | 0.66 | 0.89(0.48,1.67) | 0.70 | 0.67(0.33,1.43) | 0.29 |
| Q4 | 0.36(0.15,0.82) * | 0.02* | 1.03(0.36, 2.97) | 0.95 | 1.03(0.46,2.34) | 0.93 | 0.77(0.27,2.23) | 0.61 |
| P for trend | 0.018* |  | 0.97 |  | 0.98 |  | 0.58 |  |

Table 3 Odds ratio (95%CI) for the associations between lean body mass and four cognitive tests, stratified by sex ^a^

|  | DSST impairment | | CARED-WL impairment | | CARED-DR impairment | | AF impairment | |
| --- | --- | --- | --- | --- | --- | --- | --- | --- |
|  | female | male | female | male | female | male | female | male |
| Model 1^c^ | 0.95(0.91, 0.99) * | 0.98(0.94, 1.02) | 0.98(0.95, 1.01) | 0.98(0.96,1.00) * | 1.00(0.96,1.04) | 0.99(0.97,1.00) | 0.99(0.96,1.01) | 0.99(0.97,1.02) |
| Model 2^d^ | 0.90(0.84, 0.96) * | 0.96(0.91, 1.00) | 0.98(0.93, 1.03) | 0.97(0.93,1.01) | 0.97(0.93,1.02) | 0.98(0.94,1.03) | 0.97(0.91,1.03) | 0.98(0.95,1.02) |

Table 4 Odds ratio (95%CI) for the associations between lean body mass and cognitive tests separated by sex, by predicted lean body mass index quartiles ^a^

| Lean body mass | DSST impairment | | | CARED-WL impairment | | CARED-DR impairment | | | AF impairment | | |
| --- | --- | --- | --- | --- | --- | --- | --- | --- | --- | --- | --- |
|  | female | male | female | | male | | female | male | | female | male |
| Q1 | ref | ref | ref | | ref | | ref | ref | | ref | ref |
| Q2 | 0.65(0.34, 1.22) | 0.55(0.13, 2.29) | 1.25(0.74, 2.11) | | 9.22(0.91,93.47) | | 1.07(0.6421.78) | 3.74(0.41,33.74) | | 1.01(0.62,1.65) | 1.37(0.20,9.43) |
| Q3 | 0.87(0.28, 2.74) | 0.29(0.09, 0.98) * | 0.80(0.29, 2.22) | | 5.94(0.63,55.91) | | 0.52(0.23,1.16) | 2.93(0.45,19.20) | | 0.86(0.37,2.04) | 0.88(0.18,4.28) |
| Q4 | 0.19(0.04, 0.88) | 0.24(0.07, 0.86) * | 2.43(0.45,13.13) | | 6.21(0.69,55.53) | | 1.41(0.39,5.12) | 3.39(0.47,24.61) | | 0.39(0.15,1.03) | 1.00(0.15,6.66) |
| P for trend | 0.26 | 0.03* | 0.57 | | 0.69 | | 0.61 | 0.80 | | 0.48 | 0.72 |
